# A chemoproteoinformatics approach demonstrates that aspirin increases sensitivity to MEK inhibition by directly binding to RPS5

**DOI:** 10.1093/pnasnexus/pgac059

**Published:** 2022-05-16

**Authors:** Motoki Watanabe, Shogen Boku, Kaito Kobayashi, Yoichi Kurumida, Mamiko Sukeno, Mitsuharu Masuda, Katsura Mizushima, Chikage Kato, Yosuke Iizumi, Kiichi Hirota, Yuji Naito, Michihiro Mutoh, Tomoshi Kameda, Toshiyuki Sakai

**Affiliations:** Department of Molecular-Targeting Prevention, Kyoto Prefectural University of Medicine, 602-8566 Kyoto, Japan; Department of Molecular-Targeting Prevention, Kyoto Prefectural University of Medicine, 602-8566 Kyoto, Japan; Cancer Treatment Center, Kansai Medical University Hospital, 573-1010 Osaka, Japan; Artificial Intelligence Research Center, National Institute of Advanced Industrial Science and Technology (AIST), 135-0064 Tokyo, Japan; Artificial Intelligence Research Center, National Institute of Advanced Industrial Science and Technology (AIST), 135-0064 Tokyo, Japan; Department of Drug Discovery Medicine, Kyoto Prefectural University of Medicine, 602-8566 Kyoto, Japan; Department of Molecular-Targeting Prevention, Kyoto Prefectural University of Medicine, 602-8566 Kyoto, Japan; Department of Human Immunology and Nutrition Science, Kyoto Prefectural University of Medicine, 602-8566 Kyoto, Japan; Department of Endocrine and Breast Surgery, Kyoto Prefectural University of Medicine, 602-8566 Kyoto, Japan; Department of Molecular-Targeting Prevention, Kyoto Prefectural University of Medicine, 602-8566 Kyoto, Japan; Department of Human Stress Response Science, Institute of Biomedical Science, Kansai Medical University, 573-1010 Osaka, Japan; Department of Human Immunology and Nutrition Science, Kyoto Prefectural University of Medicine, 602-8566 Kyoto, Japan; Department of Molecular-Targeting Prevention, Kyoto Prefectural University of Medicine, 602-8566 Kyoto, Japan; Artificial Intelligence Research Center, National Institute of Advanced Industrial Science and Technology (AIST), 135-0064 Tokyo, Japan; Department of Drug Discovery Medicine, Kyoto Prefectural University of Medicine, 602-8566 Kyoto, Japan

**Keywords:** chemical biology, molecular dynamics simulations, ribosomal protein S5, MEK inhibitor, acetylsalicylic acid (aspirin)

## Abstract

MEK inhibitors are among the most successful molecularly targeted agents used as cancer therapeutics. However, to treat cancer more efficiently, resistance to MEK inhibitor-induced cell death must be overcome. Although previous genetic approaches based on comprehensive gene expression analysis or RNAi libraries led to the discovery of factors involved in intrinsic resistance to MEK inhibitors, a feasible combined treatment with the MEK inhibitor has not yet been developed. Here, we show that a chemoproteoinformatics approach identifies ligands overcoming the resistance to cell death induced by MEK inhibition as well as the target molecule conferring this resistance. First, we used natural products, perillyl alcohol and sesaminol, which induced cell death in combination with the MEK inhibitor trametinib, as chemical probes, and identified ribosomal protein S5 (RPS5) as their common target protein. Consistently, trametinib induced cell death in RPS5-depleted cancer cells via upregulation of the apoptotic proteins BIM and PUMA. Using molecular docking and molecular dynamics (MD) simulations, we then screened FDA- and EMA-approved drugs for RPS5-binding ligands and found that acetylsalicylic acid (ASA, also known as aspirin) directly bound to RPS5, resulting in upregulation of BIM and PUMA and induction of cell death in combination with trametinib. Our chemoproteoinformatics approach demonstrates that RPS5 confers resistance to MEK inhibitor-induced cell death, and that aspirin could be repurposed to sensitize cells to MEK inhibition by binding to RPS5.

Significance Statement Our study emphasizes the power and potential of chemoproteoinformatics screening to discover novel cancer drugs and their targets. First, chemoproteomics, using chemical probes that induced cell death in combination with the MEK inhibitor, demonstrated that ribosomal protein S5 (RPS5) confers this resistance. Next, chemoinformatics including molecular dynamics (MD) simulations to screen RPS5-binding ligands led to the discovery of aspirin, which sensitized MEK inhibitor-treated cells to cell death. Thus, our findings provide a novel insight into the mechanism of resistance to cell death induced by MEK inhibition and suggest that our supercomputer-based in silico screening for RPS5-binding ligands has the potential as a platform for drug discovery (including repurposed drugs like aspirin) to increase sensitivity to the MEK inhibitor.

## Introduction

The MEK inhibitor has been established as the standard treatment of *BRAF*-mutated cancers including melanoma (1), lung carcinoma (2, 3) and thyroid carcinoma (4). However, the efficacy of the MEK inhibitor for other malignancies, notably those with *RAS* mutation, appears to be insufficient (5–7), although RAS activates a major downstream MAPK signaling.

We discovered and developed the first-in-class and best-in-class MEK inhibitor trametinib by the screening of p15 inducers, which reactivated retinoblastoma (RB) protein (8, 9). Generally, the activation of RB protein leads to arrest cell cycle and protect cells from apoptosis (10–12). Indeed, trametinib remarkably induces G_1_ arrest with dephosphorylation of RB protein rather than apoptosis (13). Thus, to increase the efficacy of the MEK inhibitor, the resistance to cell death has been a bottleneck.

The mechanisms of resistance to MEK inhibitor-induced cell death have been vigorously investigated using genomics approach so far. The comprehensive gene expression analysis or RNAi libraries led to the discovery of factors involved in intrinsic resistance to MEK inhibitors. For example, RNA-seq data revealed P-TEFb complex at enhancers is targetable to synergize with MEK inhibition (14). Microarray analysis found that mitochondrial biogenesis-related genes (15) or NRAS signaling (16) confer the intrinsic resistance to the MEK inhibitor. A pooled short hairpin RNA screen identified the Hippo pathway effector *YAP1* (17) and the antiapoptotic gene *BCL-XL* (18) as molecules involved in the resistance to MEK inhibitor-induced cell death. These approaches could find the target molecules and give fruitful insights about the mechanisms responsible for this resistance, which does not always lead to discover the agents that could induce cell death in combination with the MEK inhibitor. In this study, we first adopted a chemoproteomics to identify the target molecule conferring resistance to MEK inhibitor-induced cell death using natural products as chemical probes. We subsequently performed chemoinformatics including molecular dynamics (MD) simulations to screen the ligands to overcome this resistance. Thus, our interdisciplinary study including cancer biology, chemical biology, bioinformatics analyses, and computational structural biology, successfully led to the discovery of the agent and its previously unknown target to induce cell death in combination with the MEK inhibitor.

## Results

### Perillyl alcohol and sesaminol increase MEK inhibitor-induced cell death

**Fig. 1.**
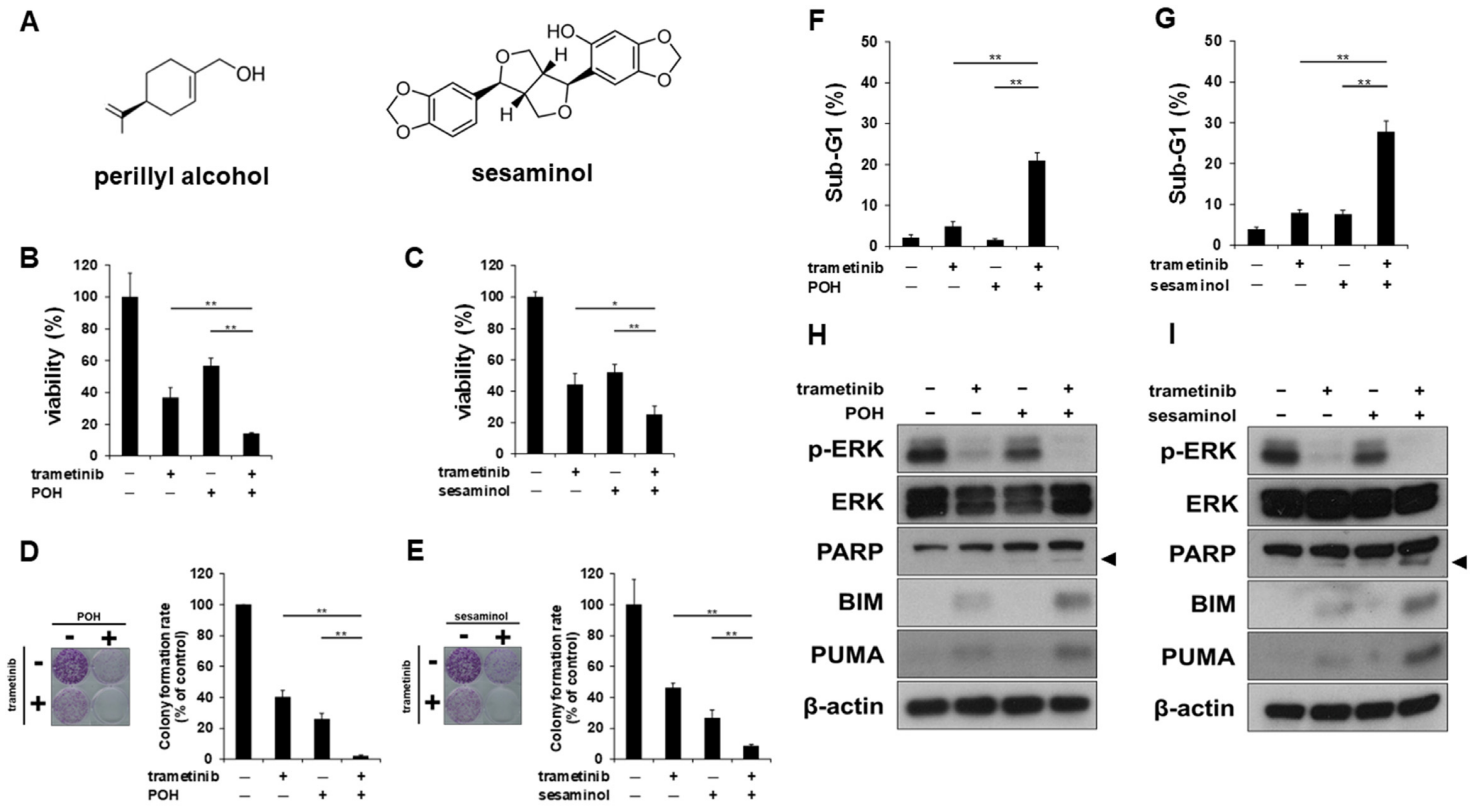
POH and sesaminol increase MEK inhibitor-induced cell death. HCT116 cells were treated with 5 nM trametinib ± 0.5 mM POH or 50 μM sesaminol. (A) Chemical structures of POH and sesaminol. (B) and (C) Growth of cells treated with trametinib ± (B) POH or (C) sesaminol. Cell viability was measured by Cell Counting Kit-8 assay after combined drug treatment for 72 h. (D) and (E) Colony formation of cells treated with trametinib ± (D) POH or (E) sesaminol. Colonies were fixed and stained with crystal violet. Representative images of stained colonies are shown (left panel). Colony numbers are shown in the graph (right panel). (F) and (G) Analyses of sub-G1 population of cells treated with trametinib ± (F) POH or (G) sesaminol. After treatment for 72 h, DNA contents of cells were determined by flow cytometry. Percentages of cells in the sub-G1 population are shown. (H) and (I) Expressions of apoptotic proteins and activities of ERK and PARP in cells treated with trametinib ± (H) POH or (I) sesaminol. Expressions of BIM and PUMA, phosphorylation of ERK, and cleavage of PARP were analyzed by western blotting after treatment for 48 h. The arrowhead indicates the cleaved form of PARP. β-Actin was used as a loading control. Bars represent means ± SD (*n* = 3).^**^P < 0.01, *P < 0.05 vs. control.

We first found that the cotreatment with the MEK inhibitor trametinib and natural products, dietary monoterpene perillyl alcohol (POH) or the sesame lignan sesaminol (Fig. 1A) suppressed both cell growth (Fig. 1B and C) and colony formation (Fig. 1D and E) in HCT116 human colorectal cancer cells with *RAS* mutation. Although trametinib alone induced little cell death, the combination treatment led to accumulation of sub-G1 cells (Fig. 1F and G) with the cleavages of PARP (Fig. 1H and I), indicative of induction of apoptosis. Consistent with this, the combinations of these natural products with trametinib upregulated BIM and PUMA (Fig. 1H and I), which play critical roles in apoptosis induced by MEK inhibition (19). These results demonstrate that POH and sesaminol overcome MEK inhibitor-induced cell death.

### POH and sesaminol directly binds to RPS5

**Fig. 2.**
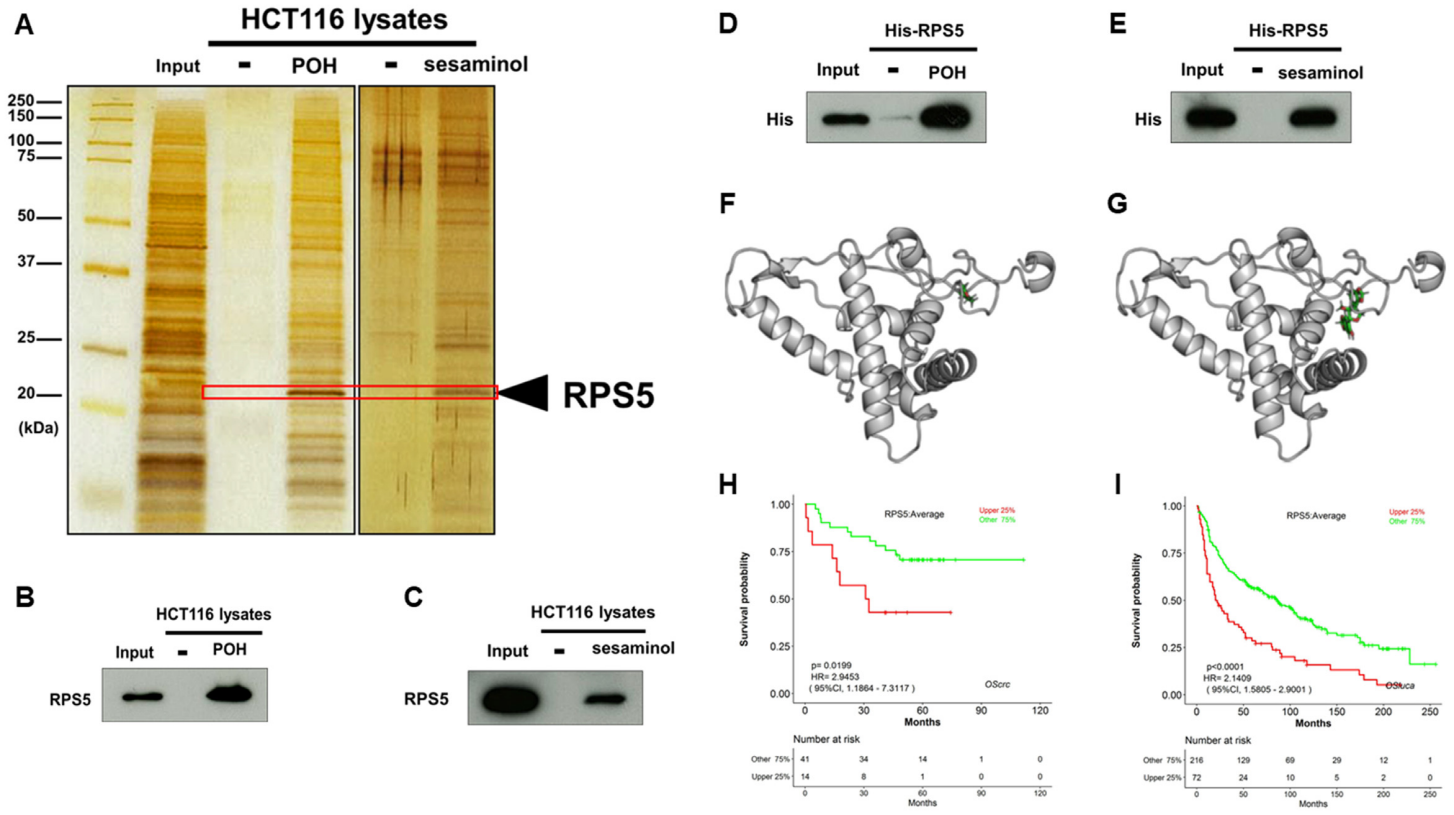
POH and sesaminol directly binds to RPS5. (A) Identification of the common binding protein of POH and sesaminol. Purified binding proteins from whole-cell extracts of HCT116 cells incubated with POH or sesaminol-immobilized FG beads were detected by mass spectrometry following silver staining. (B) and (C) Validation of RPS5 binding to (B) POH- or (C) sesaminol-immobilized FG beads. Bound RPS5 was detected by western blotting with an anti-RPS5 antibody. (D) and (E) Validation of binding of recombinant RPS5 to (D) POH- or (E) sesaminol-immobilized FG beads. Purified recombinant His-RPS5 was incubated with FG beads bearing the compounds, and bound His-RPS5 was detected by western blotting with an anti-His antibody. (F) and (G) The candidate complex structures of RPS5 and (F) POH or (G) sesaminol. Structures were generated by molecular docking simulation and the candidate structure with the highest RF-score was selected. (H) and (I) Prognostic relevance of RPS5 expression in patients with (H) colorectal or (I) lung cancer. Correlation between high expression of RPS5 (upper 25%) and overall survival rates was analyzed in patients with colorectal (GSE17537) or lung (GSE30219) cancer using data from LOGpc. Kaplan–Meier curves were generated using LOGpc. Survival curves were compared by log-rank test.

Next, we immobilized POH and sesaminol onto nanomagnetic beads (Supplementary Material Appendix, Figure S1A) to identify their target proteins in cells. After incubating these beads with whole-cell extracts of HCT116 cells, we purified POH- or sesaminol-binding proteins, as shown by silver staining (Fig. 2A). We focused on an apparent common band (∼20 kDa) and subjected the gel strip to matrix-assisted laser desorption/ionization time-of-flight mass spectrometry (MALDI-TOF MS) analysis. The strip contained peptide fragments of ribosomal protein S5 (RPS5), as confirmed by western blotting of eluates of POH- or sesaminol-binding proteins (Fig. 2B and C). We further confirmed that purified recombinant His-tagged RPS5 protein bound to POH- or sesaminol-immobilized beads (Fig. 2D and E), indicating that these compounds directly bind to RPS5. Additionally, we carried out the molecular docking simulation between RPS5 and POH or sesaminol and chose the candidate complex structures by the machine learning-based scoring system RF-Score (20) (Fig. 2F and G, respectively). Interestingly, both POH and sesaminol bound to the nonexposed-side of RPS5 in ribosome complex, suggesting that these compounds target RPS5 existing alone and affect its extraribosomal function in this context. Notably, according to LOGpc, a web-based tool providing gene expressions and survival information, high expression of RPS5 is significantly associated with poorer outcome in colorectal cancer (hazard ratio [HR], 2.9453; 95% CI, 1.1864 to 7.3117; *P* = 0.0199; Fig. 2H) and lung cancer (HR, 2.1409; 95% CI, 1.5805 to 2.9001; *P* < 0.0001; Fig. 2I) (21), suggesting that RPS5 plays an oncogenic role.

### Depletion of RPS5 overcomes resistance to MEK inhibitor-induced cell death

**Fig. 3.**
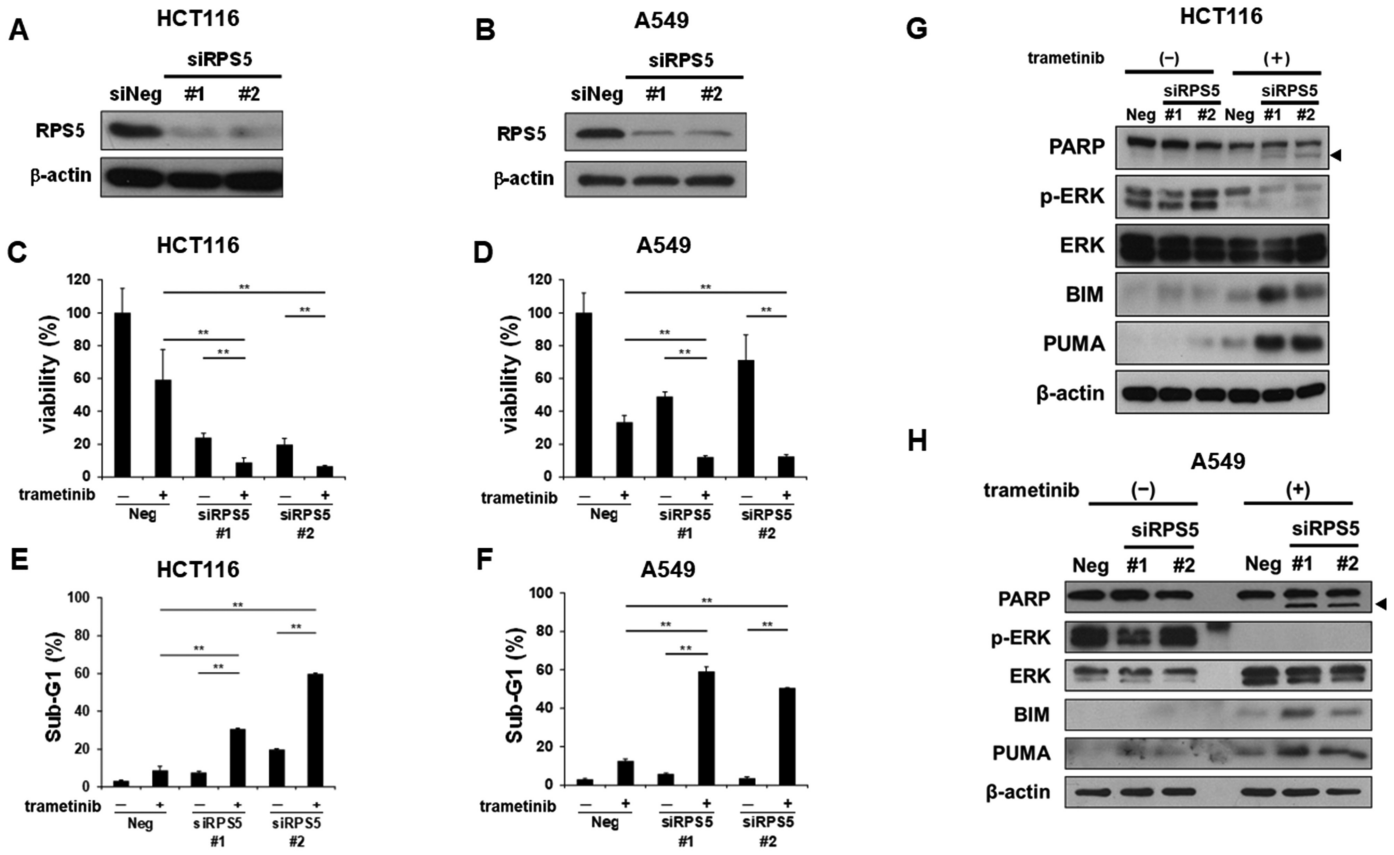
Depletion of RPS5 overcomes resistance to MEK inhibitor-induced cell death. (A) and (B) Knockdown efficacy of siRPS5. Depletion of RPS5 was validated by western blotting after 48 h (HCT116 cells) or 24 h (A549 cells) siRNA transfection in (A) HCT116 and (B) A549 cells. β-Actin was used as a loading control. (C) and (D) Effects of trametinib on growth in RPS5-depleted (C) HCT116 and (D) A549 cells. Cell viability of siRNA-transfected cells was measured by Cell Counting Kit-8 assay after 72 h trametinib treatment at 5 nM (HCT116 cells) or 1 μM (A549 cells). (E) and (F) Analyses of sub-G1 population of RPS5-depleted (E) HCT116 and (F) A549 cells treated with trametinib. After 48 h trametinib treatment at 5 nM (HCT116 cells) or 1 μM (A549 cells), DNA contents of the cells were determined by flow cytometry. Percentages of cells in the sub-G1 population are shown. (G) and (H) Expressions of apoptotic proteins and the activities of ERK and PARP in RPS5-depleted (G) HCT116 and (H) A549 cells treated with trametinib. Expressions of BIM and PUMA, phosphorylation of ERK, and cleavage of PARP were analyzed by western blotting in cells after 48 h (HCT116 cells) or 24 h (A549 cells) trametinib treatment at 5 nM (HCT116 cells) or 1 μM (A549 cells). The arrowhead indicates the cleaved form of PARP. β-Actin was used as a loading control. Bars represent means ± SD (*n* = 3). **P < 0.01 vs. DMSO with negative control siRNA (siNeg).

To determine whether RPS5 is involved in sensitivity to MEK inhibition, we knocked down RPS5 using siRNA in HCT116 cells and A549 human lung cancer cells harboring *RAS* mutation (Fig. 3A and B). In both cell lines, trametinib suppressed cell growth in RPS5-depleted cells to a greater extent than in cells transfected with negative control siRNA (Fig. 3C and D). Consistent with this, sub-G1 population synergistically increased when RPS5-depleted cells were treated with trametinib (Fig. 3E and F). Furthermore, we observed PARP cleavage and upregulation of BIM and PUMA in RPS5-depleted cells treated with trametinib (Fig. 3G and H), mirroring the events that occurred when cells were treated with trametinib and POH (Fig. 1H) or sesaminol (Fig. 1I). These results demonstrate that RPS5 confers the resistance to MEK inhibitor-induced cell death.

### P53 is required for the induction of MEK inhibitor-induced cell death in RPS5*-*depleted cells

**Fig. 4.**
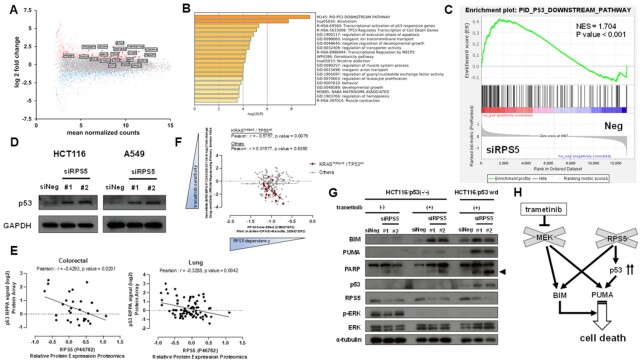
P53 is required for the induction of MEK inhibitor-induced cell death in RPS5-depleted cells. (A) Differential gene expression analysis between HCT116 cells after 48 h siRPS5 transfection and those of negative control siRNA. In MA plot, upregulated and downregulated genes in RPS5-depleted HCT116 cells are indicated in blue and red, respectively. Gene set in PID_P53_DOWNSTREAM_PATHWAY is colored in magenta, and upregulated p53-related gene symbols are indicated in square boxes. (B) The top 20 pathways of enrichment analysis with upregulated genes in RPS5-depleted HCT116 cells. (C) Enrichment plot of PID_P53_DOWNSTREAM_PATHWAY gene set in RPS5-depleted HCT116 cells with normalized enrichment score (NES) and *P*-value. (D) The expression of p53 in RPS5-depleted HCT116 and A549 cells. The expression of p53 was analyzed by western blotting in cells after 48 h (HCT116 cells) or 24 h (A549 cells) siRNA transfection. GAPDH was used as a loading control. (E) Correlation between RPS5 and p53 protein expressions using DepMap analysis. Scatter plots depict the expression of RPS5 (*x*-axis: relative protein expressions by proteomics) and p53 (*y*-axis: RPPA signal (log2) by protein array) in colorectal (*n* = 29) and lung cancer (*n* = 74) cell lines. (F) Correlation between survival dependency on RPS5 and the sensitivity to trametinib using DepMap analysis. Scatter plots depict the survival dependency on RPS5 (*x*-axis: RPS5 gene effect (DEMETER2) applied to RNAi screening datasets) and the sensitivity to trametinib (*y*-axis: log2-fold change of drug sensitivity) in *KRAS*-mutant with *p53* wild type cell lines (*n* = 20, red plots) and the other cell lines (*n* = 286, gray plots). (G) Expressions of apoptotic proteins and the activities of ERK and PARP in RPS5-depleted HCT116 and HCT116 p53(-/-) cells treated with trametinib. Expressions of BIM, PUMA and p53, phosphorylation of ERK, and cleavage of PARP were analyzed by western blotting in cells after 48 h trametinib treatment at 5 nM. The arrowhead indicates the cleaved form of PARP. α-Tubulin was used as a loading control. (H) Schematic representation of the mechanisms of cell death induction in RPS5-depeleted cells with mutant *KRAS* and wild type *p53* treated with trametinib.

To investigate how depletion of RPS5 expression enhanced trametinib-induced cell death, we carried out RNA-seq analysis using RPS5-depleted HCT116 cells. We observed that genes related to p53 downstream pathway (including *p53* target gene *PUMA* also known as *BBC3*) were most upregulated in RPS5-depleted cells compared with negative control cells (Fig. 4A–C). We then validated that depletion of RPS5 upregulated p53 protein in both HCT116 and A549 cells (Fig. 4D). For a more comprehensive analysis, we used the Cancer Dependency Map (DepMap; https://depmap.org/portal/) (22) and observed a significant inverse correlation between the protein expression of RPS5 and p53 in a panel of 29 colorectal cancer cell lines (Pearson correlation = −0.4293, *P* < 0.05) and 74 lung cancer cell lines (Pearson correlation = −0.3288, *P* < 0.01; Fig. 4E). Furthermore, the sensitivity to trametinib was inversely correlated with RPS5 dependency in survival of *KRAS*-mutant with *p53* wild type cells (*n* = 20, Pearson correlation = −0.5757, *P* < 0.01),  while no such trend was observed for the other types of cancer (*n* = 286; Fig. 4F), suggesting that p53 induction is associated with trametinib-induced cell death in RPS5-depleted cells. Next, to clarify whether p53 induction by RPS5 depletion is required for this cell death, we performed western blotting using HCT116 p53(-/-) cells. We observed only little cleavage of PARP and induction of PUMA in RPS5-depleted HCT116 p53(-/-) cells treated with trametinib as compared with those in HCT116 *p53* wild type cells, while BIM were upregulated in RPS5-depleted cells treated with trametinib regardless of *p53* status (Fig. 4G). Furthermore, using HCT-15 human colorectal cancer cells harboring *p53* mutation, we observed that sub-G1 population did not increase when RPS5-depleted HCT-15 cells were treated with trametinib (Supplementary Material Appendix, Figure S2A and B). We, therefore, concluded that trametinib induced p53-dependent cell death in RPS5-depleted cells and the induction of PUMA could be a prerequisite for this mechanism (Fig. 4H).

### A chemoinformatics approach identifies RPS5*-*binding acetylsalicylic acid as a sensitizer of MEK inhibitor-induced cell death

**Fig. 5.**
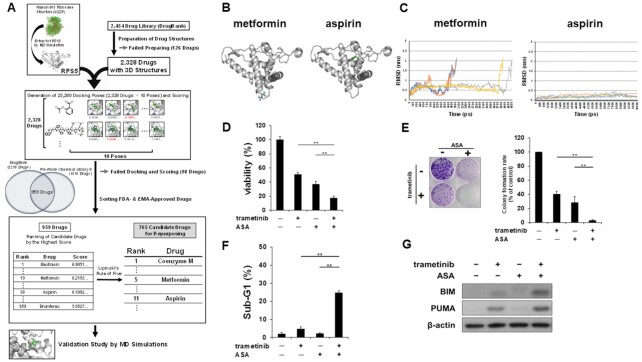
A chemoinformatics approach identifies RPS5-binding ASA as a sensitizer of MEK inhibitor-induced cell death. (A) Virtual screening for discovery of RPS5-binding drugs. Protein structures and drug structures were downloaded from RCSB PDB and DrugBank, respectively. Docking poses were generated using the Smina software and scored using RF-Score. The listed compounds were selected to focus on FDA- and EMA-approved drugs in the Prestwick Chemical Library and further narrowed down using the Lipinski's rule of five. Finally, we performed MD simulations to validate the interaction of each hit compound with RPS5. (B) The candidate complex structures of RPS5 and metformin or ASA. Structures were generated by molecular docking simulation and the candidate structure with the highest RF-score was selected. (C) Root-mean-square difference (RMSD) of metformin and ASA from initial conformation. RMSD values were compared from the initial positions for four 10 ns simulations (metformin).and five 10 ns ones (ASA). (D) Growth of cells treated with trametinib ± ASA. HCT116 cells were treated with 5 nM trametinib ± 2 mM ASA. Cell viability was measured by Cell Counting Kit-8 assay after 72 h treatment. (E) Colony formation of cells treated with trametinib ± ASA. HCT116 cells were treated with 5 nM trametinib ± 2 mM ASA. Colonies were fixed and stained with crystal violet. Representative images of stained colonies are shown (left panel). Colony numbers are shown in the graph (right panel). (F) Analyses of sub-G1 population of cells treated with trametinib ± ASA. HCT116 cells were treated with 5 nM trametinib ± 2 mM ASA for 72 h. After treatment, the DNA contents of the cells were determined by flow cytometry. Percentages of cells in the sub-G1 population are shown. (G) Expressions of apoptotic proteins in cells treated with trametinib ± ASA. HCT116 cells were treated with 5 nM trametinib ± 2 mM ASA. Expressions of BIM and PUMA were analyzed by western blotting after 24 h treatment. β-Actin was used as a loading control. Bars represent means ± SD (*n* = 3).^**^P < 0.01 vs. control.

To find more effective and feasible drugs that bind to RPS5, we carried out in silico repurposing of existing drugs via virtual screening (Fig. 5A). First, we derived the structure of the RPS5 protein from the human ribosome 80S subunit structure (PDB code: 6qzp) and existing drug structures from the DrugBank library of 2,328 drugs (23). Next, to generate structures of the complexes between RPS5 and each drug, we performed molecular docking simulation using the Smina software (24) and collected the top 10-ranked complex structures for each drug. These structures were evaluated by RF-Score (20) to rescore a “best match” RPS5-binding affinity ranking again (Supplementary Material Appendix, Table S1). In view of the feasibility of drug repurposing, we removed dietary supplements and experimental drugs and narrowed down the library list to 959 FDA- and EMA-approved drugs, referring to Prestwick Chemical Library (Supplementary Material Appendix, Table S2). Furthermore, as it is convenient and practical in a clinical setting to select an oral drug as a concomitant medication with trametinib, we then further applied Lipinski's rule of five to the screened library and found two notable drugs among 765 narrowed-down candidates: 5th ranked metformin and 11th ranked acetylsalicylic acid (ASA, also known as aspirin; < 1.5% of the final library list; Fig. 5A; Supplementary Material Appendix, Table S3), both of which has been expected to be used as a potent repurposed drug for anticancer treatment and chemoprevention (25).

To further validate the microscopic state of the interaction between RPS5 and metformin or ASA, we carried out molecular docking simulations and chose the RPS5-metformin or ASA complex structure with the highest RF-Score (Fig. 5B). To validate this prediction, we investigated the stability of the complex structure by MD simulation, in which 10 ns simulations were carried out at 300 K and 1 atm (Supplementary Material Appendix, Videos S1 and S2). In all five trajectories, root-mean-square difference (RMSD) values of ASA were maintained at low levels (Fig. 5C, right panel), suggesting that ASA stayed in its initial position and bound to RPS5, possibly because two positively charged amino acids (lysine 63 and arginine 71) located near the binding pocket of RPS5, and the side chains of these residues could form salt bridges to the carboxyl group of ASA. Contrarily, metformin dissociated from initial binding site within 5 ns in all trajectories (Fig. 5C, left panel), suggesting that the interaction between metformin and RPS5 is weak and temporary. We, therefore, served ASA as a potent candidate to further examine with trametinib.

To provide proof of concept for our approach, we next investigated whether ASA would affect sensitivity to MEK inhibition. ASA sensitized the inhibition of cell growth (Fig. 5D) and colony formation (Fig. 5E) of HCT116 cells treated with trametinib. Furthermore, treatment of HCT116 cells with trametinib and ASA synergistically induced cell death (Fig. 5F) in association with upregulation of BIM and PUMA (Fig. 5G). Next, because ASA is hydrolyzed into salicylic acid (SA) in human tissues, we investigated whether SA also bound to RPS5. In these experiments, we used the SA derivative 5-aminosalicylic acid (5-ASA) as a chemical probe and immobilized it onto nanomagnetic beads (Supplementary Material Appendix, Figure S1B). We successfully confirmed that 5-ASA-immobilized beads pulled down His-RPS5 (Supplementary Material Appendix, Figure S3A), suggesting that SA also interacts directly with RPS5, although the RPS5-binding affinity ranking of SA was lower than that of ASA (Supplementary Material Appendix, Table S1). Indeed, treatment of A549 cells with trametinib and SA synergistically induced cell death (Supplementary Material Appendix, Figure S3B) concomitant with upregulation of BIM and PUMA (Supplementary Material Appendix, Figure S3C). Taken together, these results demonstrate that ASA could sensitize cells to MEK inhibitor-induced cell death via direct interaction with RPS5 and inhibition of its function.

## Discussion

In this study, while genomics approach has been widely spread and a standard strategy to find the driver mutations or altered gene expressions in cancer, we undertook a chemoproteoinformatics approach and obtained the following novel findings: (i) a chemoproteomics, using natural products POH and sesaminol as chemical probes, revealed the molecular mechanisms how RPS5 confers resistance to cell death induced by MEK inhibition in cells with mutated *RAS* and intact *p53*; (ii) a chemoinformatics to screen RPS5-binding ligands led to the discovery of an old drug aspirin, which sensitized MEK inhibitor-treated cancer cells to cell death. Thus, our interdisciplinary approach through this two-series of screening could identify RPS5, which is not a driver gene product but targetable, with aspirin as its ligand at one scope to increase the efficacy of the MEK inhibitor with induction of cell death (Supplementary Material Appendix, Figure S4).

We first found that POH and sesaminol directly bound to RPS5 and induced cell death in combination with trametinib. POH is a dietary monoterpene derived from the mevalonate pathway in some herbs and plants and has been expected to be developed as a chemopreventive and chemotherapeutic agent against cancer (26). Although POH has been reported to inhibit various oncogenic pathway, including RAS (27–29), NF-κB (30), and AP-1 signaling (31), and upregulate cyclin-dependent kinase inhibitors including p15 (32), p21 (32, 33), and p27 (33), to our knowledge, the present study is the first report showing the direct target of POH in cancer cells. Sesaminol, an extract from sesame oil, is a metabolite from the shikimate pathway via the phenylpropanoid pathway (34). We recently identified the mitochondrial inner membrane protein adenine nucleotide translocase 2 as the other target protein of sesaminol (35). Although the structure and synthetic pathway of POH and sesaminol are different from each other, it is interesting that both of those bound to RPS5, the deletion of which consistently phenocopied the effects of POH and sesaminol on the sensitivity to MEK inhibitor-induced cell death.

Other important findings taken from this study is the discovery that RPS5 negatively regulates the expressions of p53 and plays an antiapoptotic role in cancer cells. Conversely, it has been reported that some RPs including RPL5 (36, 37), RPL11 (37–39), RPL23 (36, 40), and RPS7 (41) interact with MDM2 or HDM2, an E3 ubiquitin ligase for p53 protein, and stabilize p53 protein, suggesting that these RPs play a tumor suppressor role. Thus, the relationship between extraribosomal functions and p53 expression are controversial, and further studies will be required to determine more precisely the mechanism(s) how RPS5 regulates the expressions of p53 and a proapoptotic protein such as BIM, which was upregulated independently of p53 in the context of our study (Fig. 4G and H).

We also found that in combination with MEK inhibition, ASA directly targets RPS5 to induce cell death in HCT116 cells, which do not express cyclooxygenase-2 (COX-2) (42), a canonical target of ASA. Although ASA exerts COX-independent tumor suppressive effect (43–45), only one previous study has reported a direct target of ASA other than COX with the prediction of a complex molecular structure: Dai et al. identified heparinase as a direct target of ASA that inhibited cancer metastasis and angiogenesis (46). Interestingly, we suggested that the active metabolite of ASA, SA also interacted directly with RPS5 using the affinity beads immobilizing 5-ASA as a chemical probe (Supplementary Material Appendix, Figures S1B and S3A). Thus, ASA could be a feasible and safe candidate to exhibit the combined efficacy with the MEK inhibitor in various tissue and organ even after being hydrolyzed.

Finally, our study has some limitations. First, neither the sensitivity nor the specificity of the molecular docking simulation is sufficient. Specifically, although POH and sesaminol were shown to directly bind to RPS5 by a pull-down assay using magnetic beads and recombinant RPS5 protein (Fig. 2D and E, respectively), these compounds showed relatively low RPS5-binding affinity (Supplementary Material Appendix, Table S1). On the other hands, metformin, which was ranked 5th among 765 compounds in the docking simulation (Fig. 5A; Supplementary Material Appendix, Table S3), failed to maintain binding to RPS5 (Fig. 5C; Supplementary Material Appendix, Video S1). Thus, the molecular docking simulation has a limitation to identify bona fide candidates although it can make a quick and simple prediction, and we believe that this problem could be compensated by adding MD simulations, which could analyze the dynamic processes of the binding on timescales. Second, to validate our proof of concept, we performed in vivo experiments employing the HCT116 xenograft mice model orally treated with trametinib and/or aspirin; however, daily administration of a sufficient dose of aspirin (100 mg/Kg, i.g.) did not reduce tumor volumes at all, while trametinib (2 mg/Kg, i.g. every other day) resulted in an approximately 50% reduction in the tumor growth (data not shown). These results imply that the absorption, metabolism, or distribution of aspirin was impaired in this physiological setting. Interestingly, Gut microbes in mice, such as *Lysinibacillus sphaericus*, degrades aspirin before absorption and dampens its chemopreventive efficacy (47). Thus, as the bioavailability of aspirin is dependent on gut microbe in mice, it may be difficult to adequately assess the efficacy of aspirin in in vivo experiments using tumor-bearing mice. Nevertheless, considering that aspirin has been retrospectively shown to reduce the risk of various types of cancer (48), the efficacy of aspirin in combination with the MEK inhibitor may be evaluated by additional approaches including a real-world data analysis of patients prescribed with trametinib with or without regular use of aspirin.

In summary, our chemoproteomic approach to identifying the targets of natural products led to the discovery of RPS5, which is not considered oncogenic and may be unreachable by genetic sequencing focusing on genetic alterations including driver mutations. More importantly, by combining a chemoinformatics approach with MD simulation, we obtained results that not only expanded our biochemical understanding of the mechanisms of resistance to MEK inhibitor-induced cell death, but also allowed the rapid discovery of a repurposed drug that could overcome this resistance. Thus, as protein landscapes are increasingly used to understand tumorigenesis and find novel cancer therapeutic targets that are not driver gene products (49), our chemoproteoinformatics approach may provide novel opportunities for screening and validating RPS5-binding ligands for use as cancer therapeutics in combination with the MEK inhibitor in the postgenome era.

## Material and methods

### Reagents

Trametinib was obtained from Selleck Biotech (Tokyo, Japan). (S)-(-)-POH was obtained from Wako (Osaka, Japan). Sesaminol was obtained from Nagara Science (Gifu, Japan). ASA was obtained from Sigma (St. Louis, MO, USA). 5-Aminosalicylic acid (5-ASA) was obtained from Cayman Chemical (Ann Arbor, MI, USA). SA was obtained from Nacalai Tesque (Kyoto, Japan). All reagents were dissolved in dimethyl sulfoxide (DMSO) to make stock solutions. Purified recombinant human RPS5 (ab137146) was obtained from Abcam (Cambridge, UK).

### Cell lines and culture

The human colorectal cancer cell line HCT116, HCT-15, and the human lung cancer cell line A549 were obtained as NCI-60 cell lines from the NCI Developmental Therapeutics Program. HCT116 p53(-/-) cells were kindly provided by Dr B. Vogelstein (50). All cell lines were confirmed to be negative for mycoplasma infection using the MycoAlert Mycoplasma Detection Kit (Lonza Rockland, ME, USA). HCT116, HCT116 p53(-/-), and A549 cells were cultured in Dulbecco's modified Eagle's medium (DMEM) supplemented with 10% fetal bovine serum (FBS), 4 mM glutamine, 50 U/ml penicillin, and 100 μg/ml streptomycin. HCT-15 cells were cultured in RPMI-1640 supplemented with 10% FBS, 2 mM glutamine, 50 U/ml of penicillin, and 100 μg/ml of streptomycin. Cells were incubated at 37°C in a humidified atmosphere containing 5% CO_2_.

### Cell viability assay

The number of viable cells was measured using Cell Counting Kit-8 assay (Dojindo, Kumamoto, Japan). Briefly, cells were seeded at a density of 2,500 cells (HCT116 cells) or 2,000 cells (A549 cells) per well in 96-well plates. After cells were incubated with each agent or siRNA, the kit reagent WST-8 was added to the medium, and the sample was incubated for 4 h. Absorbance at 450 nm was measured using a multiplate reader (Molecular Devices, San Jose, CA, USA).

### Colony formation assay

Cells were seeded at a density of 2,000 cells per well in 6-well plates. Seeded cells were incubated for 24 h in 6-well plates and treated with the indicated agents. After incubation for an additional 2 weeks, the cells were fixed with 10% formalin and stained with 0.1% crystal violet. The area of stained colonies was quantified using ImageJ from the US NIH (Bethesda, MD, USA, https://imagej.nih.gov/ij/).

### Detection of apoptosis

Cells were seeded at a density of 50,000 cells (HCT116 and HCT-15 cells) or 25,000 cells (A549 cells) per well in 6-well plates. Cells treated with the indicated agent or siRNA were harvested by trypsinization. After centrifugation, the cells were suspended in PBS containing 0.1% Triton X-100 and 25 μg/ml propidium iodide. Stained cells were analyzed on a FACSCalibur (Becton Dickinson, Franklin Lakes, NJ, USA), and the data were analyzed using the CellQuest software (Becton Dickinson).

### Protein isolation and western blotting

Cells were lysed with buffer containing 50 mM Tris-HCl, 1% SDS, 1 mM dithiothreitol (DTT), and 0.43 mM 4-(2-aminoethyl) benzenesulfonyl fluoride hydrochloride (ABSF). The lysates were sonicated and centrifuged at 20,400 *g* at 4°C for 20 min, and the supernatants were collected. Equal amounts of protein extract were subjected to SDS-PAGE and transferred to a PVDF membrane (EMD Millipore, Billerica, MA, USA). Primary antibodies were as follows: rabbit anti-p44/42 MAPK (ERK1/2) (#9102), rabbit antiphospho-p44/42 MAPK (ERK1/2) (#9101), rabbit anti-PARP (#9542), rabbit anti-Puma (#4976), rabbit anti-p53 (#9282), mouse anti-His-Tag monoclonal antibody (#2366; Cell Signaling Technology, Beverly, MA, USA), rabbit anti-BIM (ab32158), rabbit anti-RPS5 (ab58345, Abcam), mouse anti–β-actin monoclonal antibody (A5441, Sigma), mouse anti-GAPDH (#5G4, HyTest Ltd., Turku, Finland), and mouse antihuman α-tubulin monoclonal antibody (CP06, Calbiochem, La Jolla, CA, USA). Signals were detected with Chemi-Lumi One L (Nacalai Tesque) or Immobilon Western Chemiluminescent HRP Substrate (EMD Millipore).

### Preparation of the reagent-fixed beads

Magnetic FG beads were purchased from Tamagawa Seiki (Nagano, Japan). The protocol of fixation of POH onto the beads is now in submission. Fixation of sesaminol onto beads with epoxy linkers (TAS8848N1110) was performed as previously described (34). Briefly, the beads were incubated at 60°C for 24 h with 100 mM sesaminol in DMF containing potassium carbonate. The next day, after washing with DMF, the beads were washed with Milli-Q water. To fix 5-ASA onto FG NHS (N-hydroxysuccinimide) beads (TAS8848N1141), the beads were incubated at room temperature overnight with 30 mM 5-ASA in absolute DMF. The next day, after washing with absolute DMF, the beads were masked with 1 M ethanolamine in absolute DMF. The beads were then washed with aqueous methanol solution (50% v/v) and Milli-Q water.

### Purification and identification of POH and sesaminol-binding proteins

HCT116 cells were lysed with binding buffer [50 mM Tris-HCl, 150 mM NaCl, 1% NP-40, 1 mM DTT, and 0.43 mM ABSF] at 4°C for 30 min and then centrifuged. The supernatants were used as whole-cell extracts of HCT116 cells. The extracts were incubated with agent-immobilized or empty beads at 4°C for 4 h and then washed three times with binding buffer. Bound proteins were eluted with Laemmli dye and subjected to SDS-PAGE. Proteins were stained with aqueous AgNO_3_, and each strip containing a protein was cut out and digested with Sequencing Grade Modified Trypsin (Promega, Madison, WI, USA). After in-gel digestion, the peptide fragments from each strip were analyzed using an Autoflex II mass spectrometer (Bruker Daltonics, Billerica, MA, USA).

## RNA-Seq

Total RNA was isolated from cells treated with each siRNA using Sepasol-RNA I (Nacalai Tesque) according to the manufacturer's instructions and processed using a TruSeq Stranded mRNA sample prep kit (Illumina, San Diego, CA, USA). Poly(A) RNA libraries were then constructed using a TruSeq Stranded mRNA library preparation kit (Illumina) and sequenced in 100-bp paired-end reads on an Illumina NovaSeq6000 platform. Sequencing data were deposited into the DNA Data Bank of Japan Sequence Read Archive (accession nos. DRR356490 to DRR356498). RNA-Seq was performed in triplicate.

## Transcriptomics analysis

RNA-Seq reads were quantified using ikra (v.2.0.1) (51), an RNA-Seq pipeline centered on Salmon (52). Ikra automated the RNA-Seq data-analysis process, including the quality control of reads sra-tools v.2.10.9, Trim Galore v.0.6.7 (53) using Cutadapt v.3.2 (54), and transcript quantification (Salmon v.1.4.0, using reference transcript sets in GENCODE release 37 for humans), and tximport v.1.6.0). These tools were used with default parameters. Count tables were imported into integrated differential expression and pathway analysis (iDEP v.0.95), an integrated web application for gene ontology (GO) analysis of RNA-Seq data (55). Quantified transcript reads were filtered at a threshold of 0.5 counts per million (CPM) in at least one sample and transformed using EdgeR (3.36.0): log2(CPM + c), with a pseudocount of 4. The enrichment analysis was performed in iDEP using fold-change values returned by DESeq2 (1.34.0). Absolute fold changes > 2 were considered enriched and investigated further by Metascape (56). Gene set enrichment analysis (GSEA) was performed by using the Broad Institute GSEA software (v.4.2.3) (57). *P*-values < 0.05 were used to determine which functions could be used for further investigation.

### Bioinformatics analysis

Kaplan–Meier curves were generated using the LOGpc (Long-term Outcome and Gene Expression Profiling Database of pan-cancers) from the Biomedical Informatics Institute (http://bioinfo.henu.edu.cn/Index.html). The LOGpc tool was used to analyze the association between RPS5 expression and prognosis, using the upper quartile value to dichotomize RPS5 profiles of patients. The dataset GSE17537 provided cancer prognosis and microarray data including RPS5 expression profiles of colorectal cancer samples. Survival data and RPS5 expression profiles for patients with lung cancer were acquired from the GSE30219 dataset (20). Survival curves were compared using a log-rank test.

Datasets described as follows were obtained from the DepMap website (https://depmap.org/portal/): (i) Protein expressions of RPS5 (RPS5 (P46782) relative protein expression proteomics) and p53 (RPPA signal (log2) protein array) in colorectal and lung cancer cell lines (ii) Survival dependency on RPS5 (RPS5 gene effect (DEMETER2) RNAi) and the sensitivity to trametinib (trametinib (BRD: BRD-K12343256-001-08-9) log2 fold change Drug sensitivity (PRISM Repurposing Primary Screen) 19Q4). Pearson correlation coefficients were used to evaluate each correlation.

### RNAi

Oligonucleotides for siRNA targeting RPS5 were obtained from Thermo Fisher Scientific (Waltham, MA, USA). The following siRNAs were used: siRPS5 #1 (HSS109357; Stealth siRNAs), 5“-GGAGCACCGAUGAUGUGCAGAUCAA-3”; siRPS5 #2 (HSS184431; Stealth siRNAs), and 5'- GCCGCAACAACGGCAAGAAGCUCAU-3'; negative control siRNA (12,935,112; Stealth RNAi siRNA Negative Control Med GC Duplex #2). Only RNA sequences of sense strands are shown. Cells were transfected with 10 nM siRNA using the Lipofectamine RNAiMAX Reagent (Invitrogen, Carlsbad, CA, USA). Cells were treated with each siRNA for 6 h, and the medium was then replaced with fresh medium or one containing each agent.

### In silico drug screening

The structure of RPS5 was derived from the 6 ribosome 80S subunit structure (PDB code: 6qzp). To relax the structure under aqueous conditions, we carried out a 500 ns MD simulation (see detail in the next section, “Molecular dynamics simulation”); the last structure was used for molecular docking calculations. Existing drug structures were derived from the library prepared in DrugBank, which contains 2,328 drugs approved by the US FDA (23). First, to generate complex structures, molecular docking simulation was performed using the Smina software (24), and the complex structures with the top 10 binding scores were collected for each drug. This docking simulation was done for the entire surface of protein. Next, these structures were re-evaluated by rf-score (20), and the top-ranking complex structure was selected. Finally, the listed compounds were selected to focus on FDA- and EMA-approved drugs in Prestwick Chemical Library and further narrowed down using the Lipinski's rule of five.

### MD simulations

The microscopic states of interactions between RPS5 and drug compounds (metformin and ASA) were studied by MD simulation. The structure of RPS5 was derived from the 6 ribosome 80S subunit structure (PDB code: 6qzp). The top-ranking complex structure of RPS5 with each compound, as determined based on rf-score (20), was used as the initial structure for MD simulation. A total of 10 ns simulations were performed four times (metformin) and five times (ASA). RPS5 was described the AMBERff14SB force field (58). Metformin and ASA were also described using the GAFF force field (59) with restrained electrostatic potential (RESP) charges (60). The atomic information for metformin and ASA is shown in mol2 format in Supplementary Material Appendix, Tables S4 and S5, respectively. Water molecules were described using the TIP3P model (61). The system contained a protein (RPS5), a metformin or an ASA molecule, 19,802 water molecules, and 39 sodium and chloride ions corresponding to a 150 mM solution. To neutralize the net charge of the system, 15 (for metformin) or 13 (for ASA) chloride ions were added. These molecules were placed in a dodecahedral box with a side length of 96.4 Å. The temperature was maintained at 300 K using Langevin dynamics, and a pressure of 1 atm was maintained using the Parinello–Rahman barostat (62). MD simulations were conducted using the GROMACS 2020.3 simulator (63). The figures were generated using VMD (64).

### Statistical analysis

All quantitative data are presented as means ± standard deviation (SD). Significance was evaluated by two-tailed unpaired Student's t test. A value of *P* < 0.05 was considered to indicate a significant difference relative to the indicated control.

## Supplementary Material

pgac059_Supplemental_Files

## Notes

^1^ M.W. and S.B. contributed equally to this work.

**Competing Interest:** T.S. reports receiving patent fee regarding trametinib from the JT Pharmaceutical Institute. No potential conflicts of interest were disclosed by the other authors.
