# Supplementary material for: A chemoproteoinformatics approach demonstrates that aspirin increases sensitivity to MEK inhibition by directly binding to RPS5
Source: PNAS Nexus. 2022 May 16;1(2):pgac059. doi: 10.1093/pnasnexus/pgac059 (PMC9802315; doi:10.1093/pnasnexus/pgac059)
Supplement: pgac059_Supplemental_Files [file pgac059_supplemental_files.zip › PNASNEXUS-PNASNEXUS-2021-00178-s04.pdf]

```
@MOLECULE
MHM
   20    19     1     0     0
SMALL
No Charge or Current Charge


@ATOM
      1 N1           3.5400     1.4200     0.0000 nh          1 MHM
-1.006723
      2 H1           3.9220     0.5540    -0.3270 hn          1 MHM
0.387466
      3 H2           2.5390     1.4120    -0.0540 hn          1 MHM
0.387466
      4 C3           3.9900     1.7680     1.2270 c2          1 MHM
1.093289
      5 N2           3.1500     2.5260     1.9620 n2          1 MHM
-0.996920
      6 H3           3.6450     3.1670     2.5460 hn          1 MHM
0.446941
      7 N4           5.2400     1.4100     1.6050 nh          1 MHM
-1.012598
      8 H4           5.7680     0.8980     0.9290 hn          1 MHM
0.418326
      9 C6           5.7760     1.5210     2.8410 c2          1 MHM
1.064754
     10 N5           4.9930     1.8850     3.8770 n2          1 MHM
-0.990870
     11 H5           5.5280     2.0270     4.7130 hn          1 MHM
0.409991
     12 N8           7.0730     1.1940     3.1080 nh          1 MHM
-0.425010
     13 C9           7.9820     1.3140     2.0290 c3          1 MHM
-0.165011
     14 H9           9.0230     1.2990     2.4220 h1          1 MHM
0.094480
     15 H10          7.8940     0.4850     1.2910 h1          1 MHM
0.108046
     16 H11          7.8610     2.2960     1.5180 h1          1 MHM
0.104763
     17 C7           7.1790    -0.0550     3.7720 c3          1 MHM
-0.312259
     18 H6           8.2330    -0.2420     4.0770 h1          1 MHM
0.102955
     19 H7           6.5790    -0.0800     4.7070 h1          1 MHM
0.125649
     20 H8           6.8450    -0.8910     3.1150 h1          1 MHM
0.165265
@BOND
     1     2     1 1
     2     3     1 1
     3     4     1 1
     4     5     4 2
```

```
     5     6     5 1
     6     7     4 1
     7     8     7 1
     8     9     7 1
     9    10     9 2
    10    11    10 1
    11    12     9 1
    12    13    12 1
    13    14    13 1
    14    15    13 1
    15    16    13 1
    16    17    12 1
    17    18    17 1
    18    19    17 1
    19    20    17 1
@MHMSTRUCTURE
     1 MHM          1 TEMP                   0 ****  ****    0 ROOT
```
