# Supplementary material for: A chemoproteoinformatics approach demonstrates that aspirin increases sensitivity to MEK inhibition by directly binding to RPS5
Source: PNAS Nexus. 2022 May 16;1(2):pgac059. doi: 10.1093/pnasnexus/pgac059 (PMC9802315; doi:10.1093/pnasnexus/pgac059)
Supplement: pgac059_Supplemental_Files [file pgac059_supplemental_files.zip › PNASNEXUS-PNASNEXUS-2021-00178-s05.pdf]

```
@MOLECULE
ASA
   20    20     1     0     0
SMALL
No Charge or Current Charge


@ATOM
      1 O4           3.5400     1.4200     0.0000 o           1 ASA
-0.654319
      2 C12          4.5560     2.1240     0.0000 c           1 ASA
0.892680
      3 C13          5.8920     1.4410    -0.0010 c3          1 ASA
-0.521041
      4 H18          5.7490     0.3610    -0.0010 hc          1 ASA
0.142322
      5 H19          6.4490     1.7330     0.8890 hc          1 ASA
0.142322
      6 H20          6.4490     1.7330    -0.8900 hc          1 ASA
0.142322
      7 O1           4.5280     3.5580     0.0000 os          1 ASA
-0.382605
      8 C5           3.2650     4.2420     0.0000 ca          1 ASA
0.326602
      9 C6           3.2670     5.7200     0.0000 ca          1 ASA
-0.124877
     10 C11          4.5300     6.4680    -0.0000 c           1 ASA
0.846090
     11 O2           5.7510     5.8010     0.0000 o           1 ASA
-0.794764
     12 O3           4.5080     7.7030     0.0000 o           1 ASA
-0.794764
     13 C8           2.1180     6.3930     0.0010 ca          1 ASA
-0.139694
     14 H15          2.1280     7.4830     0.0010 ha          1 ASA
0.152377
     15 C10          0.8440     5.6790     0.0010 ca          1 ASA
-0.196520
     16 H17         -0.0940     6.2340     0.0010 ha          1 ASA
0.118269
     17 C9           0.8320     4.3490     0.0010 ca          1 ASA
-0.150597
     18 H16         -0.1180     3.8140     0.0010 ha          1 ASA
0.123576
     19 C7           2.0910     3.6040     0.0000 ca          1 ASA
-0.327717
     20 H14          2.0720     2.5150     0.0000 ha          1 ASA
0.200338
@BOND
     1     2     1 2
     2     3     2 1
     3     4     3 1
     4     5     3 1
```

```
     5     6     3 1
     6     7     2 1
     7     8     7 1
     8     9     8 ar
     9    10     9 1
    10    11    10 1
    11    12    10 1
    12    13     9 ar
    13    14    13 1
    14    15    13 ar
    15    16    15 1
    16    17    15 ar
    17    18    17 1
    18    19    17 ar
    19    20    19 1
    20    19     8 ar
@SUBSTRUCTURE
     1 ASA         1 TEMP              0 ****  ****    0 ROO
```
