# Supplementary material for: A chemoproteoinformatics approach demonstrates that aspirin increases sensitivity to MEK inhibition by directly binding to RPS5
Source: PNAS Nexus. 2022 May 16;1(2):pgac059. doi: 10.1093/pnasnexus/pgac059 (PMC9802315; doi:10.1093/pnasnexus/pgac059)
Supplement: pgac059_Supplemental_Files [file pgac059_supplemental_files.zip › PNASNEXUS-PNASNEXUS-2021-00178-s06.docx]

**Supplementary Information Appendix**

**A** **chemoproteoinformatics approach demonstrates that aspirin increases sensitivity to MEK inhibition by directly binding to RPS5**

Motoki Watanabe^1^*, Shogen Boku*, Kaito Kobayashi, Yoichi Kurumida, Mamiko Sukeno, Mitsuharu Masuda, Katsura Mizushima, Chikage Kato, Yosuke Iizumi, Kiichi Hirota, Yuji Naito, Michihiro Mutoh, Tomoshi Kameda^1^ and Toshiyuki Sakai

1. Co-corresponding Authors

Motoki Watanabe: mtkw@koto.kpu-m.ac.jp

Tomoshi Kameda: kameda-tomoshi@aist.go.jp

* These authors contributed equally to this work.

This file includes Supplementary Figures S1-S4 and legends.

**A**

**B**

**Figure S1. Scheme for fixation of sesaminol or 5-aminosalicylic acid (5-ASA) onto magnetic FG beads. (*A* and *B*)** Immobilized **(*A*)** sesaminol or **(*B*)** 5-ASA on magnetic FG beads and the estimated structure of beads harboring each compound.

**A B**

**Figure S2. The MEK inhibitor does not induce cell death in *p53*-mutated RPS5-depleted cell. (A)** Knockdown efficacy of siRPS5. Depletion of RPS5 was validated by western blotting after 48 h siRNA transfection in HCT-15 cells. α-Tubulin was used as a loading control. **(B)** Analyses of sub-G1 population of RPS5-depleted HCT-15 cells treated with trametinib. After 48 h trametinib treatment at 500 μM, DNA contents of the cells were determined by flow cytometry. Percentages of cells in the sub-G1 population are shown. N.S., not significant.

**
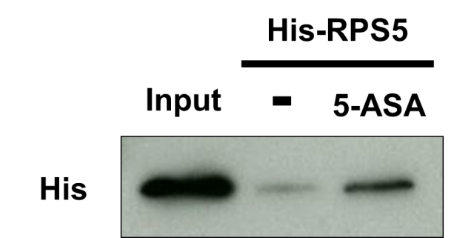
A B**

**C**


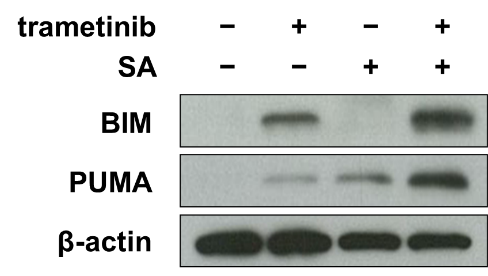


**Figure S3. Salicylic acid (SA) directly binds to RPS5 and synergistically induces cell death in combination with trametinib treatment.** **(*A*)** Validation of binding of recombinant RPS5 to 5-aminosalicylic acid (5-ASA)-immobilized FG beads. Purified recombinant His-RPS5 was incubated with 5-ASA-immobilized FG beads, and bound His-RPS5 was detected by western blotting with an anti-His antibody. **(*B*)** Analyses of sub-G1 population of cells treated with trametinib ± SA. A549 cells were treated with 1 µM trametinib ± 5 mM SA for 48 h. After treatment, DNA contents of cells were determined by flow cytometry. Percentages of cells in the sub-G1 population are shown. **(*C*)** Expression of apoptotic proteins in cells treated with trametinib ± SA. A549 cells were treated with 1 µM trametinib ± 5 mM SA. Expression of BIM and PUMA was analyzed by western blotting after 24 h treatment. β-Actin was used as a loading control. Bars represent means ± SD (n = 3). **P < 0.01 vs. control.

**Figure S4. A scheme of the chemoproteoinformatics screening.** This two-series of screening could identify the target molecule and its ligand at one scope to increase the efficacy of the MEK inhibitor with induction of cell death.

**Table S1. Affinity scores of RPS5-binding drugs, as determined by RF-Score.** Affinity scores of perillyl alcohol (POH) and sesaminol are appended and marked in red letters.

**Table S2. Affinity scores of RPS5-binding FDA- and EMA-approved drug.** The affinity score of acetylsalicylic acid (aspirin) are marked in red letters.

**Table S3. Ranking of RPS5-binding FDA- and EMA-approved drugs selected by Lipinski's rule of five.** The affinity score of acetylsalicylic acid (aspirin) are marked in red letters.

**Table S4. Atomic information of metformin model used for molecular dynamics simulation (MD) in mol2 format.**

**Table S5. Atomic information of acetylsalicylic acid (ASA) model used for molecular dynamics (MD) simulation in mol2 format.**

**Video S1. Molecular dynamics (MD) trajectory of the RPS5-metformin complex.**

**Video S2. Molecular dynamics (MD) trajectory of the RPS5-acetylsalicylic acid (ASA) complex.**
